# Pediatric Blood Cultures and Antibiotic Resistance: An Overview

**DOI:** 10.1007/s12098-019-03123-y

**Published:** 2019-12-21

**Authors:** Chand Wattal, Neeraj Goel

**Affiliations:** Department of Clinical Microbiology & Immunology, Sir Ganga Ram Hospital, Rajinder Nagar, New Delhi, India

**Keywords:** Pediatric blood culture, PICU, AMR, Etiology, Surveillance

## Abstract

Bloodstream infections (BSI) due to multidrug-resistant organisms, especially from pediatric intensive care units (PICU), are being increasingly reported across the world. Since BSI is associated with high mortality, it is essential to treat these infections early with appropriate antibiotics. Surveillance of etiology and emerging antimicrobial resistance (AMR) is considered an important step in the formulation of antibiotic policy for early treatment and judicious use of antibiotics. In this review on etiology and its antibiogram in community acquired BSI, *S. typhi* followed by *S. paratyphi A* were the major bacterial isolates. Quinolone resistance of more than 90% in *Salmonella* is now reported from all over India. Ceftriaxone remains the drug of choice for enteric fever due to its 100% susceptibility. In PICU there is an emergence of candidemia due to non-albicans candida which are now predominant isolates at few centers. BSI due to gram-negative bacteria, mostly by *Klebseilla pneumoniae* and gram-positive cocci (*S. aureus*) are the other major pathogens commonly observed in BSI from PICU. There is a high prevalence of antimicrobial resistance to commonly used antibiotics like ampicillin (94.9%–90.7%), cefotaxime (92.4%–71.4%), piperacillin-tazobactum (31.2%–27.5%) and levofloxacin (42.4%–39.8%). Resistance to carbapenems, primarily due to bla_NDM_ is seen in all the centers and the rate varies between 1%- 79% with *K. pneumoniae* and *A. baumannii* showing the maximum resistance. This review highlights the magnitude of the AMR in the pediatric population and calls for the urgent implementation of antimicrobial stewardship programs to save the remaining antimicrobials.

## Introduction

Multi-drug-resistant (MDR) infections in the pediatric age group, especially due to gram-negative bacteria (GNB) are increasing world over with higher mortality [1]. Overall mortality in Indian PICU due to hospital-acquired infections (HAIs) has been estimated to be 26%. Bloodstream infections (BSI) are considered as the most serious infections in pediatric intensive care units (PICU) and carry the highest mortality with an estimated attributable mortality of 3% and crude mortality of 18% [2, 3]. Many risk factors, especially in PICU, for HAIs are common for adults and children which include exposure to invasive devices including intravascular catheters, intubation, hyper-alimentation, and other comorbidities like immune-suppression. Additional risk factors in the pediatric population include immature innate and adaptive immunity which further affects the severity and duration of infections [4, 5]. Infectious Diseases Society of America (IDSA) guidelines on Antimicrobial stewardship program (AMSP) recommends the provision of institute specific etiology and antibiogram for various HAIs for formulating appropriate antibiotic policy for effective treatment within first few hours to decrease the mortality with BSI [6]. In spite of the above fact, there is scarce data available from India on the etiology of BSI and its susceptibility pattern in pediatric population.

## Surveillance of HAIs

Surveillance in a pediatric healthcare facility is usually dependent on the national, regional or institutional health requirements, along with the commitment and resources available. Information technology (IT) support is recognized as an essential important resource to generate reliable HAI surveillance data [6]. The patchy data on the incidence of HAIs in developing countries is mostly due to lack of national or regional AMR surveillance network, compared to the western countries [4]. In India AMR data is plagued by the absence of major National AMR surveillance network. Earlier initiatives included Indian Clinical Epidemiological Network (INCLEN) & Indian Network for Surveillance of Antimicrobial Resistance (INSAR) [7, 8]. More recently, the Indian Council of Medical Research (ICMR) has launched a national level the Anti-Microbial Resistance Surveillance and Research Network (AMRSN) across the country in 2013 for generating data on HAIs and AMR, but has not stratified the data as per the age [9]. However, resistance patterns can significantly differ in adults and children [10], therefore this data cannot be extrapolated to the pediatric age group. Therefore as of now, we have patchy institution-specific data available for assessing the etiology and AMR data. The authors here have done the review of isolates from pediatrics patients from the year 2014 through 2018. This document will review the literature from other centers from India, as well. In this review, only pediatric blood culture isolates will be discussed, since this is the sample that remains the most sacrosanct among the bacteriological samples sent for culture and sensitivity and remains most representative of the BSI.

## Blood Culture Positivity

The positivity rate of blood culture in pediatrics from India varies from 7.2% to 88.5% with median of 35.4% [11]. The wide variation in the positivity of blood culture can be attributed to many variables like the etiology of BSI, prior intake of antibiotics, volume and methods of blood culture practices. BSI due to endocarditis, meningitis and septic shock, are associated with high organism load as compared to other BSI, therefore such patients have high positivity as compared to BSI due to other reasons [12].

The volume of blood is an important determinant in the positivity of blood cultures. Clinical & Laboratory Standard Institute (CLSI) recommends 10 ml (adults) and 3 ml (Pediatrics) blood in two sets of two bottles each with one of the bottles in the set as an anerobic one amounting to 40 ml blood in adults and 12 ml blood in pediatrics being subjected to culture. This can detect 90–95% of bacteremia [13, 14]. Studies suggest that of the multiple 20 ml blood cultures drawn in 24 h in adults, approximately 70% will have positive cultures after the first draw, 85% at second and 97% after 3rd and 99% after fourth [15, 16]. In-spite of the above CLSI guidelines, it is a common practice in India to obtain only one blood culture bottle (5 ml) for diagnosing BSI. The authors presume increased cost maybe the hindrance in the implementation of CLSI guidelines. At authors’ institute, they have the policy to collect at least 1 set of blood cultures (2 blood cultures bottles of 5 ml each) from 2 different peripheral sites and an additional 1 blood culture bottle (5 ml) if there is a central line. This practice also aids in differentiating coagulase negative *Staphylococcus* (CONS) as colonizers or pathogens as per CDC guidelines [17].

Most modern laboratories nowadays utilize automated incubation and detection system that has higher efficiency and lower contamination rate. Most of such automated systems have a shorter incubation time to positivity as compared to the non-automated conventional systems. Addition of resins and charcoal help in improving the positivity of blood cultures provided by automated systems by absorbing/neutralizing the presence of antibiotics [18].

## Etiology of BSI/Blood Culture Isolates

**Fig. 1:**
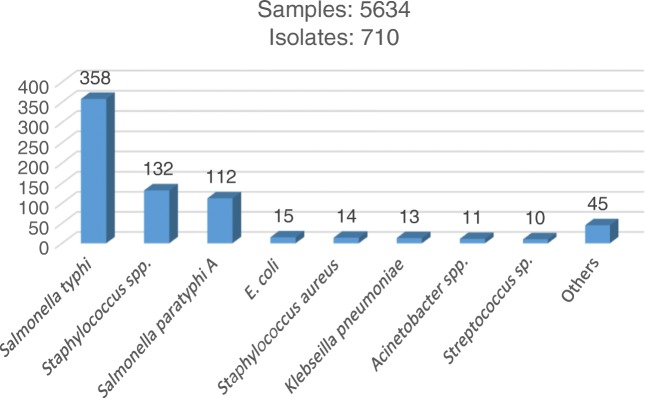
Etiology of BSI in pediatric age group in OPD samples (2014–2018)

Enteric fever in India affects children and adolescents of almost all age groups but the highest numbers of cases are reported in school going children between 5 and 15 y of age followed by preschool children (2–5 y) [19]. Enteric fever due to *S. typhi* and *Paratyphi A* remains the most common cause of community-acquired BSI in the pediatric age group at authors’ hospital (Fig. 1).

In a study by Iyer et al., the prevalence of enteric fever at a pediatric tertiary care hospital from South India during the ten-year study period from 2007 to 2016, was found to be 0.5% and 0.1% for *S. typhi* and *S. paratyphi A*, respectively [20]. On the other hand, in a systematic review and meta-analysis in adults and children in India, a prevalence of 9.7% of *S. typhi* and 0.9% of *S. paratyphi A* was observed [21]. At authors’ center too they have observed a similar higher prevalence of *S. typhi* (6.3%) compared to *S. paratyphi A* (1.98%) (Fig. 1).

MDR in Salmonella is estimated to vary between 1.9% to 4.1% [20–22]. Multiple reports from all over India have also shown improved susceptibility to ampicillin, co-trimoxazole, and chloramphenicol (ACCo) [20, 22]. Similarly, at authors’ center too they noted a low ACCo resistance of 2.3% [22]. The decline in the ACCo resistance could be attributed to limited use of these antibiotics due to the availability of better alternatives like 3rd generation cephalosporins. On the other hand, there has been an increasing trend of resistance to quinolones in Salmonella and now almost 90–100% quinolone resistance in *S. typhi* & *Paratyphi A* has been reported especially after British Society of Antimicrobial & Chemotherapy (BSAC) revised its breakpoint guidelines in the year 2011 [20]. At authors’ center, they have also observed a high resistance to quinolones (96%) and nil resistance to ceftriaxone in the pediatric population during the years 2014–2018. However, few cases of ceftriaxone resistance have been reported from Bangladesh, Nepal, United Arab Emirates and Germany [23–26]; therefore a strict vigilance on the emergence of ceftriaxone resistance in salmonella needs to be maintained. Azithromycin is recommended as an alternative therapy to ceftriaxone in the case of resistance to quinolones as per WHO guidelines and the current scarce literature on azithromycin shows little resistance to this drug [20, 27]. Therefore, nowadays, ceftriaxone and azithromycin remain the drugs of choice for the empiric treatment of enteric fever although treatment may be modified based on susceptibility report of the isolates.

**Fig. 2:**
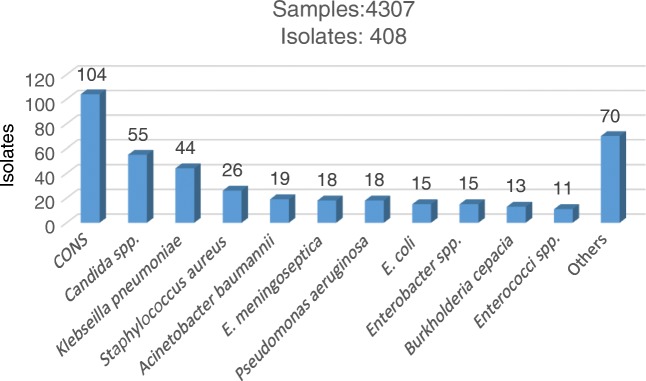
Isolates of BSI in Pediatric ICU (2014–2018)

The etiology of BSI in PICU has been changing over the last few years of authors’ surveillance. Instead of GNBs being commonly reported as the predominant bacterial isolates in many studies [4, 11, 28], now *Candida spp.* have become the predominant pathogens in the pediatric ICUs. In a 5 y study period on the etiology of BSI in PICU at Sir Ganga Ram Hospital, authors processed 4307 blood samples, out of which 408 (9.5%) isolates were obtained. CONS (25.5%), were the commonest bacteria isolated, followed by *Candida spp.* (13.5%), *Klebseilla pneumoniae* (10.8%), *Staphylococcus aureus* (6.4%), *Acinetobacter baumannii* (4.6%), *Pseudomonas aeruginosa* (18%) and *E. coli* (15%) (Fig. 2). Few other studies have also shown CONS as a common organism isolated in BSI [28, 29]. CONS are normally considered as skin contaminants but can be a cause of BSI in cases of indwelling central lines, supported by two positive cultures from two different sites accompanied by specific clinical signs and symptoms of BSI [17]. In authors’ case, they did not have adequate data to differentiate between the two. In one study CONS from a single positive culture are considered as contaminant in 75% to 95% [14]. An important finding in present study was the emergence of *Candida spp.* as the 2nd most common cause of BSI in PICU. Observed candidemia rates in authors’ setting were probably due to the greater use of broad-spectrum antibacterial agents, invasive devices, more extensive surgical procedures and the use of advanced life support in various critical and immunosuppressed patients as has been shown in a previous studies [30, 31]. Similar to authors’ findings, HAIs due to *Candida spp*. are increasingly reported worldwide and are considered as major pathogens among immunosuppressed and critically ill patients [4, 22, 29, 31].

**Fig. 3:**
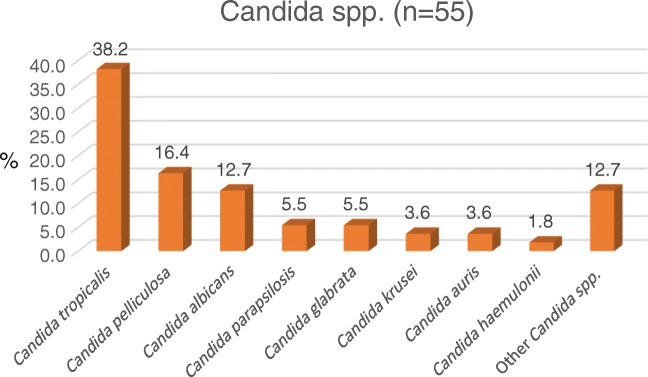
Various species of Candida isolated in BSI from PICU (2014–2018)

Additionally, there was an emergence of non albicans candidemia at authors’ centre. *C. albicans* constituted just 12.7% of the total candidemia. Most common *Candida spp*. isolated were *Candida tropicalis* (38.2%), followed by *Candida pelliculosa* (16.4%) and *Candida albicans* (12.7%) (Fig. 3). Similar to authors’ data, in an another study by Lakshmi et al., on BSI in PICU, there was a shift to non-*Candida albicans* species with the majority (77.8%) of them being *C. tropicalis* [28]. The emergence of non albicans candida at authors’ centre has been shown to correlate with increasing use of fluconazole in authors’ previous study [31].

*K. pneumoniae* was the commonest GNB isolated; the possible source could be respiratory tract infections as it was the most common isolate in pediatric pneumonia in PICU. The finding of *S. aureus* as the commonest pathogen in gram-positive cocci (GPCs) makes empirical treatment of BSI in pediatric ICU difficult as it would involve treatment against candida, GNBs and GPCs as per the etiology of authors’ PICU. One important observation in authors’ study was the presence of central lines as a major risk factor for candidemia in the PICU, and in the absence of central lines, GNB was the commonest bacterial isolate.

Other studies from Indian PICU have shown higher rates of BSI due to GNBs as compared to GPCs and *Candida spp*. [11, 29, 32]. In a retrospective study of nosocomial infections in PICU between 1994 and 2003, Singhi et al. observed GNB as the predominant isolates, common being *Klebsiella pneumoniae* (20.1%) *Enterobacter spp.* (16.6%) and *Acinetobacter spp*. (8.6%) [32]. In a study by Thacker et al., it was shown that overall *enterobacteriaceae* isolates constituted more than half of the GNBs in pediatric BSI and twice that of *Pseudomonas spp.* [33].

In another Indian study on 285 children admitted in PICU, the incidence of BSI was observed as 31.2 episodes/ 1000 patient days with the mean age of BSI as 3.7 ± 3.5 y. GNBs were the major isolates (53.5%) with *Klebsiella pneumoniae* being the most prevalent (24.4%), followed by *S. aureus* (20.9%). CONS (8.1%) and *Candida spp.* (10.5%) were the other predominant isolates in this study [28]. Dharmapalan et al., reviewed the published data of Indian Neonatal and pediatric population during 2000–2015 from India. After an extensive electronic search, 89 papers were reviewed; this 15-year data reported bacteremia caused by GNB to be around 53.3% whereas gram-positive organisms caused infection in 30.9% of the pediatric population [11].

The data from developing countries is in contrast to the western countries, where GPCs are the predominant isolates from nosocomial infections in PICU. In a National Healthcare Safety Network (NHSN) by Centers for Disease Control and Prevention from 2011 to 2014 in 1003 hospitals, 20,390 pediatric HAIs were reported. *Staphylococcus aureus* (17%), followed by CONS (17%), *Escherichia coli* (11%), *Klebsiella pneumoniae* (9%), and *Enterococcus faecalis* (8%) were the commonest organisms isolated [34].

## Emerging Antimicrobial Resistance

The authors studied the antibiogram of BSI of the important bacteria during the last five years at PICU of their tertiary care centre. Methicillin-resistant *Staphylococcus aureus* (MRSA) prevalence of 46% and clindamycin resistance of 23% was observed in 26 isolates of *S. aureus.* Similar to authors’ data, in one of the largest data from Indian Network for Surveillance of Antimicrobial Resistance (INSAR) group on the prevalence of MRSA across 15 centres from India in a mixed population of pediatrics and adults, an overall MRSA prevalence of 41% was found from a total of 26,310 *S. aureus* isolates [8]. Although the majority of the *S. aureus* isolates were from skin and soft tissue infections followed by BSI. There was significantly higher rates of resistance in MRSA (erythromycin: 70.8%, clindamycin: 46.6%, gentamicin: 58.3%) as compared to methicillin-susceptible *Staphylococcus aureus* (MSSA) (erythromycin: 26.3%, clindamycin: 14.7%, gentamicin: 17.4%). There was no documented resistance to vancomycin or teicoplanin and linezolid. High rates of MRSA (50%) among the pediatric population has also been reported from another study as well [11]. Due to the high prevalence of MRSA in PICU, it appears that vancomycin should be used for suspected GPC infection pending susceptibility reports.

In GNBs, authors’ observed a very low susceptibility of 3rd generation cephalosporins in GNBs ranging from 7% to 33% (Table 1). Such a high level of resistance to cephalosporins renders them ineffective for the empirical treatment of BSIs. Similarly, they observed a low susceptibility to beta lactam-beta lactamase inhibitors (BL-BLI) like piperacillin/tazobactum and cefoperazone/sulbactum (16% to 67%) and quinolones (13% to 64%). What is most worrisome is that even the carbapenems, considered as the last resort drugs, show low susceptibility in *A. baumannii* (21%), *K. pneumoniae* (42%), while *E. coli* (71%), and *P. aeruginosa* (67%) had relatively higher susceptibility. Fortunately, the authors have not seen much resistance to colistin in GNBs except in *K. pneumoniae* (2%) and *Enterobacter spp*. (4%).

**Table 1:** Percentage susceptibility of GNBs isolated from PICU from blood

GNBs	No. of isolates	Ampicillin	Cefuroxime	Ceftriaxone	Ceftazidime	Cefepime	Piperacillin+Tazobactum	Cefoperazone+Sulbactum	Quinolones	Gentamicin	Amikacin	Netilmicin	Ertapenem	Imipenem/Meropenem	Colistin
*E. coli*	15	0	0	0	–	6	44	44	40	60	88	60	60	71	100
*Klebseilla pneumoniae*	44	3	5	7	–	16	27	33	24	31	47	33	36	42	98
*Pseudomonas aeruginosa*	18	–	–	–	67	67	67	67	64	67	67	69	–	73	100
*Acinetobacter baumannii*	19	–	0	5	–	16	16	17	13	16	21	17	–	21	100
*Enterobacter spp.*	15	0	13	33	–	40	53	53	47	53	73	70	53	67	96

*GNB* Gram-Negative Bacteria

Similar to authors’ data, other centers are also reporting high resistance in GNBs from PICU. Dharmapalan et al., observed more than 90% resistance for ampicillin in GNBs, whereas resistance to amikacin ranged from 22.4% to 50%. Similarly, high resistance to cephalosporins of 62.6% in *K. pneumoniae* and 47.5% in *E.coli* was observed. High rates of resistance in the GNBs were also noted for piperacillin-tazobactum varying between 16.7% to 42% [11]. Other studies have also reported high resistance to commonly used first-line antibiotics, ampicillin (94.9%–90.6%%), cefotaxime (92.4%–71.4%), piperacillin-tazobactum (31.2%–27.5%) and levofloxacin (42.4%–39.8%) [35]. Since cephalosporins are the first-line antibiotics recommended in India in pediatrics practice for enteric fever, meningitis, and pneumonia [11], its high rate of resistance is worrisome on the efficacy of these antibiotics. Resistance to carbapenems too is now commonly reported from different centers in India. In an analysis of 82 published literature from different Indian NICU and PICU, a median carbapenem resistance of 1% in *K. pneumoniae,* 9% in *E. coli*, 16.7% in *P. aeruginosa* and 11.5% in *A. baumannii* was seen [11]. A similar resistance of 11.1% to imipenem was seen in *enterobacteriaceae* from tertiary care centers from South-India from years 2012 to 2014 [28]. Higher resistance to carbapenems in PICU at authors’ centre could be due to the fact that it is a tertiary care center where many critical cases are referred with high case mix index (CMI) [36], who are either already colonized with multidrug resistant organisms (MDROs) or require higher prescription of antibiotics which may contribute to the emergence of MDROs [37].

There is little data from India on the type of carbapenemase prevalence in carbapenem resistant enterobacteriaceae (CRE). The authors could only find one study from South India, Vellore on the type of carbapenemases in BSI from PICU. In this study bla _NDM_ (72.7%) was shown to be the predominant plasmid in CRE, followed by bla _OXA_ (9.1%), and a combination of bla _NDM_/bla _OXA_ (9.1%) [37]. Therefore, bla KPC has got replaced by the above two plasmids. The treatment of BSI with CRE is challenging as it is accompanied by up to 90% resistance to other drugs like amikacin [37]. Often, colistin is used as the last resort drug to treat CRE infections. But this is further complicated by the lack of robust data on pharmacokinetics/pharmacodynamics of this drug. Further, questions on the efficacy of colistin monotherapy or combination therapy are still not resolved. Therefore clinicians are forced to often use unproven therapies, including colistin, in cases of CRE which is associated with high mortality of up to 52% [37]. Expectedly, the rampant use of colistin has led to the emergence of its resistance in GNBs. A median resistance to colistin resistance from different pediatric centers across India has been shown to be as: *E.coli* (8.8%), *K. pneumoniae* (3.8%), *A. baumaanii* (0%), and *P. aeruginosa* (0%) [11]. Similarly, in adult ICUs at authors’ hospital, they observed colistin resistance only in *K. pneumoniae* BSI, albeit at a much higher rate of 17% [22]; therefore it remains a grave concern for its potential spread to PICU too. The recent increase in the resistance to colistin in GNBs across the world has been associated with the emergence of plasmid-mediated gene mcr-1 in the year 2016 [38]. Colistin resistance was previously associated with only chromosomal mutations but now it is feared that mcr-1 may emulate NDM-1 in its rapid global widespread. Colistin resistance in GNBs has resulted in the emergence of pan drug-resistant (PDR) bugs with no antibiotics left for the treatment. Although not so common in PICUs, PDR bugs are now a common scenario in adult ICUs [22]. Therefore it would be prudent to implement AMSP on an urgent basis in the PICUs to salvage whatever little is left of the remaining antibiotics.

Emergence of non-albicans *Candida spp.* at authors’ center has resulted in increased resistance to the first-line anti-fungal drug, fluconazole. Low fluconazole susceptibility of 90.5% and 47.6% was noted for *C. tropicalis* and *C. pelliculosa,* respectively from authors’ centre [39]. Even *C. albicans* which was showing 100% susceptibility to fluconazole in the year 2012 is now showing reduced susceptibility of 88.9% [39]. This reduced susceptibility was partly due to revision in the break points of fluconazole for *C. albicans* in 2012 [40]. *C. auris* is an emerging multi-drug resistant *candida spp*. in ICU settings and has shown 0%, 7.6% and 7.6%, 79.5% susceptibility to fluconazole, amphotericin, voriconazole and caspofungin, respectively, which make these difficult to treat PDR bugs [39].

To conclude, this review of BSI in the Indian pediatric population highlights that *Salmonella* continues to be the major etiology of community-acquired BSI. In such cases, empirical treatment with ceftriaxone remains the most pragmatic approach. Azithromycin can be considered as an alternative to ceftriaxone while ACCo sensitivity has returned. On the other hand, BSI related to Indian PICU is a complex scenario. The etiology can be ranging from candida to GNBs and GPCs. In the presence of central lines, it would be prudent to give antifungals empirically for the treatment of BSI, otherwise, treatment with carbapenem or combination of carbapenem and colistin is advisable due to high resistance to the first-line therapy. In the absence of a national network of AMR surveillance for pediatrics infections, institute specific hospital-based surveillance of AMR is essential for formulating antibiotic policy for the judicious use of the antibiotics.
